# Development of n-Type Porphyrin Acceptors for Panchromatic Light-Harvesting Fullerene-Free Organic Solar Cells

**DOI:** 10.3389/fchem.2018.00473

**Published:** 2018-10-09

**Authors:** Un-Hak Lee, Wisnu Tantyo Hadmojo, Junho Kim, Seung Hun Eom, Sung Cheol Yoon, Sung-Yeon Jang, In Hwan Jung

**Affiliations:** ^1^Division of Advanced Materials, Korea Research Institute of Chemical Technology, Daejeon, South Korea; ^2^Department of Chemistry, Kookmin University, Seoul, South Korea

**Keywords:** porphyrin acceptors, n-type porphyrins, organic solar cells, non-fullerene acceptors, panchromatic absorption

## Abstract

The development of n-type porphyrin acceptors is challenging in organic solar cells. In this work, we synthesized a novel n-type porphyrin acceptor, P_Zn_-TNI, via the introduction of the electron withdrawing naphthalene imide (NI) moiety at the meso position of zinc porphyrin (P_Zn_). P_Zn_-TNI has excellent thermal stability and unique bimodal absorption with a strong Soret band (300–600 nm) and weak Q-band (600–800 nm). The weak long-wavelength absorption of P_Zn_-TNI was completely covered by combining the low bandgap polymer donor, PTB7-Th, which realized the well-balanced panchromatic photon-to-current conversion in the range of 300–800 nm. Notably, the one-step reaction of the NI moiety from a commercially available source leads to the cheap and simple n-type porphyrin synthesis. The substitution of four NIs in P_Zn_ ring induced sufficient n-type characteristics with proper HOMO and LUMO energy levels for efficient charge transport with PTB7-Th. Fullerene-free organic solar cells based-on PTB7-Th:P_Zn_-TNI were investigated and showed a promising PCE of 5.07% without any additive treatment. To the best of our knowledge, this is the highest PCE in the porphyrin-based acceptors without utilization of the perylene diimide accepting unit.

## Introduction

For decades, organic solar cells (OSCs) have been studied as a portable and low-cost power generator due to their unique advantages: light-weight, use of earth-abundant organic materials, solution-processability and flexibility. The power conversion efficiency (PCE) of OSCs, an important parameter to determine the performance of the OSCs, has been continuously improved by optimization of light-harvesting in active layers and hole/electron charge transport pathways (Zhan et al., 2015; Zhao J. et al., 2016; Lin et al., 2017; Che et al., 2018; Hou et al., 2018). In the 2000s, low-bandgap polymer donors and n-type fullerene acceptors blended OSCs were developed to make broad absorption in the active layer. Polymer donors showing a strong absorption coefficient were responsible for light-harvesting and exciton generation, while the n-type fullerenes effectively separate and transfer the electrons to the electrode (Kim et al., 2006; Liang et al., 2010; Li et al., 2012; Liao et al., 2013). However, this was not enough to cover all the visible band by blending polymer donors and fullerene acceptors. In the 2010s, new types of OSCs replaced the fullerenes with strong light-harvesting organic non-fullerene acceptors (NFAs). The active layer was composed of organic donors and NFAs, called fullerene-free OSCs (Cheng et al., 2017; Lin et al., 2017; Tang et al., 2018; Yan et al., 2018). Due to the strong absorption of both NFAs and organic donors in the visible area, their complementary absorption is important to achieve panchromatic photon-to-current conversion in the active layer. There are several successful strategies for panchromatic absorption in the visible and near infra-red area. The most common approach is mixing wide-bandgap donors and low-bandgap small-molecule NFAs such as ITIC (Zhao W. et al., 2016, 2017; Lin et al., 2017; Yao et al., 2017; Zhao F. et al., 2017). Another approach is mixing low-bandgap donors and wide-bandgap NFAs (e.g., blending of PTB7-Th and perylene-diimide (PDI)-based acceptor) (Wu et al., 2016; Duan et al., 2017; Liang et al., 2017; Eom et al., 2018). The red-dye PDIs enable a strong absorption in the short-wavelength area of 400–600 nm, while showing effective n-type characteristics.

As a new approach for panchromatic absorption in an active layer, utilization of a nature-inspired porphyrin dye has recently emerged in OSCs.(Gao et al., 2015; Li et al., 2016; Hadmojo et al., 2018) The porphyrin dyes have peculiar bimodal absorption characteristics composed of Soret and Q bands; strong transition from ground state (S0) to second excited state (S2) yields the Soret band, while the weak transition from S0 to first excited state (S1) provides the Q-band. Thus, the strong Soret absorption of porphyrin dyes enables efficient short-wavelength absorption in the 400–600 nm, which can be blended with low-bandgap donors having a dominant absorption in the 600–800 nm for panchromatic absorption. In addition, the long-wavelength absorption of the Q-band intensifies the light harvesting in the low bandgap area where abundant solar flux exists. However, most of the developed artificial porphyrin derivatives are p-type materials and only a few porphyrin derivatives currently show n-type characteristics with a promising PCEs over 5% (Hadmojo et al., 2017; Zhang et al., 2017). Exploring new structures for n-type porphyrin materials is challenging in fullerene-free OSCs.

In this study, we synthesized a novel porphyrin acceptor, P_Zn_-TNI, via Sonogashira coupling of 5,10,15,20-tetrakis-ethynyl porphyrin Zinc (II) (P_Zn_) and 4-bromo-N-(2-ethylhexyl)-1,8-naphthalimide (NI). NI is easily synthesized from the commercially available 4-bromo-1,8-naphthalic anhydride. This one-step reaction is beneficial in terms of time and cost for synthesizing the star-shape molecules that require an excess of NIs. Since the NI has n-type characteristics, the substitution of NIs to the four meso positions of P_Zn_ enables the excellent n-type properties as an electron acceptor. The ethyne π-bridge unit is incorporated between NI and P_Zn_ to increase the backbone planarity. The synthesized P_Zn_-TNI showed excellent thermal stability with 5% weight loss temperature of 412°C and showed unique bimodal absorption behavior with maximum peaks at 479 nm and 719 nm. The uncovered UV-Vis absorption spectrum from P_Zn_-TNI is completely covered by the blending of a polymer donor, PTB7-Th, which resulted in the panchromatic photon-to-current conversion from 300 to 800 nm in OSCs. The planar backbone structure of P_Zn_-TNI assists the sizable face-on orientation in the PTB7-Th:P_Zn_-TNI blend film without additive treatment, which resulted in the highest PCE of 5.07% (*V*_OC_ = 0.72 V, *J*_SC_ = 13.84 mA cm^−2^, and fill factor = 0.51) in the additive-free OSCs. The excessive ordering of PTB7-Th:P_Zn_-TNI film via pyridine additive rather reduced the photovoltaic performances. Our successful utilization of NI moiety in the P_Zn_ core will broaden and diversify the synthetic approaches for developing high-efficiency porphyrin acceptors.

## Experimental

### Synthesis

5,10,15,20-tetrakis-ethynyl porphyrin Zinc (II) (3): Macrocyclic porphyrin compound 1 was synthesized according to the reported general procedure (Yen et al., 2006). Compound 1 (0.90 g, 1.29 mmol) and zinc acetate (2.4 g, 13.1 mmol) were dissolved in the co-solvent (200 ml) of dichloromethane:methanol = 9:1 v/v%. The resulting mixture was refluxed at 65°C for 24 h. After removing the solvents, the remaining solid was rinsed with dichloromethane (300 mL) to give a purple solid compound 2 (0.65 g, yield: 62%). Without further purification, compound 2 was directly used to make compound 3. Compound 2 (0.60 g, 0.79 mmol) was dissolved in 100 mL anhydrous tetrahydrofuran (THF). Tetra-n-butylammonium fluoride solution 1.0 M in THF (3.6 ml, 3.6 mmol) was slowly added to the reaction mixture. The resulting mixture was stirred at room temperature for 4 h. After removing the solvents, the crude solid product was rinsed sequentially with methanol, dichloromethane, water and acetone. After drying the dark purple solid product 3 (180 mg, yield: 49%), it was immediately used in the next step to prevent the coupling reaction between the two terminal alkynes. ^1^H NMR (THF-*d*_6_, 400 MHz, ppm): δ 9.60 (s, 2H), 5.37 (s, 1H),

*N*,*N'*-(2-hexyldecyl) 4-bromo naphthalene imide (4): 2-Hexyldecyl amine (2.4 g, 9.93 mmol) was added to the suspension of 4-bromo-1,8-naphthalic anhydride (2.5 g, 9.03 mmol) in dry ethanol (50 mL). The reaction mixture was refluxed overnight at 110°C, and then cooled down to room temperature. After evaporating the solvents, the remaining crude solid was purified using column chromatography on silica gel with an eluent of CH_2_Cl_2_:n-hexane = 4:1 to give a yellow solid compound 4 (3.0 g, 66%). ^1^H NMR (CDCl_3_, 400 MHz, ppm): δ 8.65 (d, *J* = 6.4 Hz, 1H), 8.56 (d, *J* = 7.6 Hz, 1H), 8.40 (d, *J* = 8.0 Hz, 1H), 8.03 (d, *J* = 7.6 Hz, 1H), 7.82 (m, 1H), 4.14 (t, *J* = 8.0 Hz, 2H), 1.70 (m, 1H), 1.24 (br, 27H), 0.87 (t, *J* = 7.2 Hz, 3H).

P_Zn_-TNI: Compound 3 (100 mg, 0.212 mmol), compound 4 (1.06 g, 2.12 mmol), Pd_2_(dba)_3_ (40 mg, 0.044 mmol) and AsPh_3_ (100 mg, 0.33 mmol) were dissolved in dry THF (15 mL) and triethylamine (15 mL). The reaction mixture was stirred at 65°C for 4 days under N_2_ atmosphere, and then quenched by distilled water. The organic layer was extracted using dichloromethane and water, and then the moisture in the organic solution was removed by Na_2_SO_4_. After evaporating the solvents, the solid residue was purified by column chromatography using an eluent of CH_2_Cl_2_:n-hexane = 4:1. Then it was further purified using recycling size exclusion chromatography to give a deep green solid P_Zn_-TNI (290 mg, 75%). ^1^H NMR (THF-*d*_6_, 400 MHz, ppm): δ 9.02 (br, 2H), 8.85 (br, 1H), 8.40 (d, *J* = 4.8 Hz, 1H), 8.28 (d, *J* = 5.6 Hz, 1H), 8.19 (br, 1H), 7.88 (br, 1H), 4.24 (m, 2H), 1.92 (m, 1H), 1.39 (br, 27H), 0.96 (m, 3H). ^13^C NMR (THF-*d*_6_, 100 MHz, ppm): δ 163.58, 163.32, 149.75, 131.61, 131.49, 130.46, 130.18, 128.58, 128.35, 127.90, 124.02, 123.05, 102.08, 101.88, 96.30, 41.51, 33.14, 31.09, 31.05, 31.02, 30.99, 30.92, 30.87, 30.61, 29.22, 28.66, 23.82, 14.71. MALDI-TOF-MS: m/z: calcd. for C_140_H_160_N_8_O_8_Zn: 2145.17 [M] ^+^; found 2145.994.

## Results and discussion

**Scheme 1:**
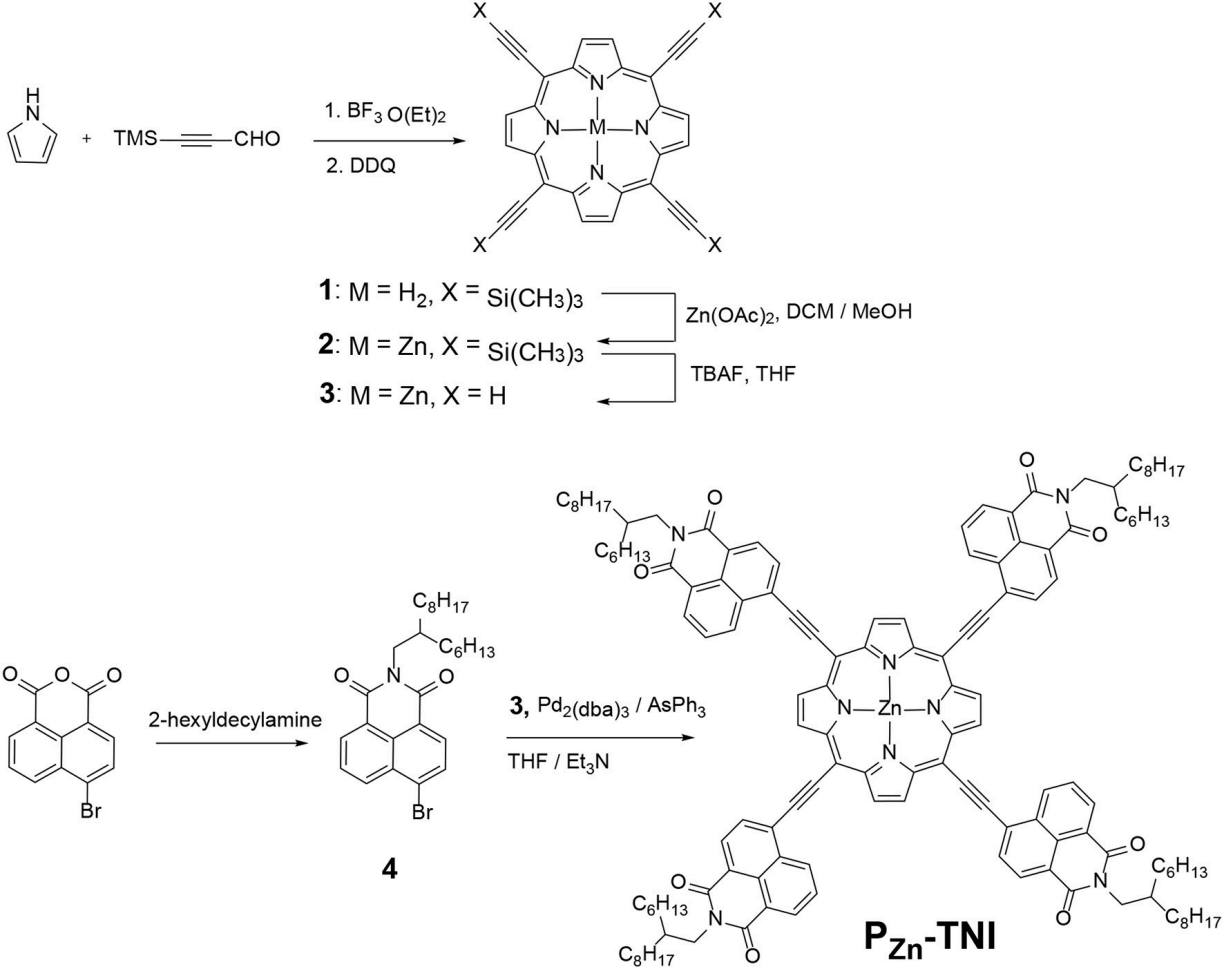
Synthetic route for n-type porphyrin acceptor, P_Zn_-TNI.

The synthetic procedure of P_Zn_-TNI was recorded in Scheme 1 and in the Supporting Information (SI) in detail. The porphyrin ring 1 was synthesized from pyrrole and 3-(trimethylsilyl)propiolaldehyde in the presence of BF_3_·Et_2_O followed by oxidation with 2,3-dichloro-5,6-dicyano-1,4-benzoquinone (DDQ). The zinc porphyrin (P_Zn_) compound 2 was obtained using Zn(OAc)_2_. The deprotection of TMS group by tetra-n-butylammonium fluoride (TBAF) was performed immediately before synthesizing the final acceptor, P_Zn_-TNI. 4-Bromo-1,8-naphthalic anhydride purchased from Sigma-Aldrich was alkylated with 2-hexyldexylamine to give a compound 4. This one-step reaction to prepare the electron withdrawing NI unit is highly beneficial in terms of time and cost for achieving the n-type porphyrins. The final porphyrin acceptor, P_Zn_-TNI, was achieved via Sonogashira coupling with P_Zn_ compound 3 and excess of NI compound 4, which was identified by ^1^H-NMR and matrix assisted laser desorption/ionization time-of-flight mass spectrometry (MALDI-TOF-MS) (Figures S1–S4). The synthesized P_Zn_-TNI showed excellent solubility in common organic solvents such as tetrahydrofuran (THF), dichloromethane (DCM), and chloroform (CF). In addition, it has excellent thermal stability, which was determined by thermal gravimetric analysis (TGA), with a 5% weight loss temperature (T_5d_) of 412°C under an N_2_ atmosphere (Figure S5).

**Figure 1:**
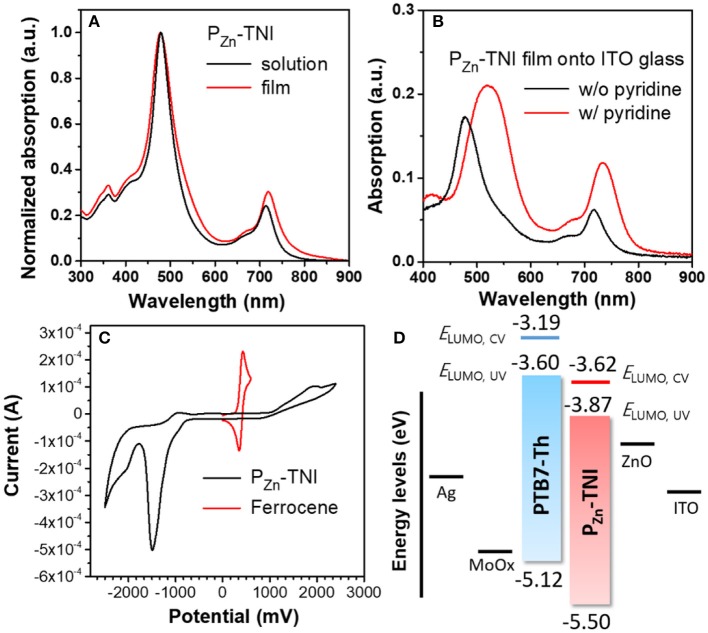
Absorption spectra of PZn-TNI **(A)** in chloroform and in the film on the quartz plate, and **(B)** on ITO glass with or without pyridine additive. **(C)** Cyclic voltammogram of PZn-TNI, and **(D)** energy diagram of the PTB7-Th:PZn-TNI device.

**Figure 2:**
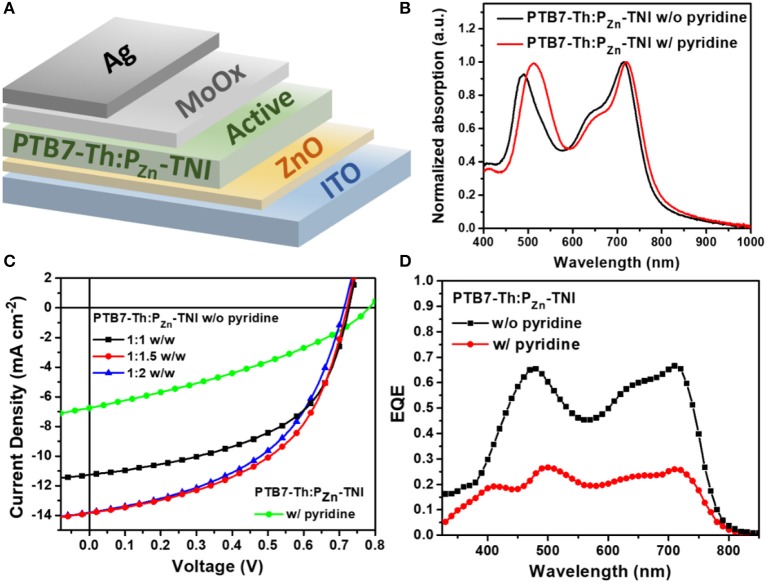
**(A)** Device structure of PTB7-Th:P_Zn_-TNI based OSCs, **(B)** absorption spectra of PTB7-Th:P_Zn_-TNI active layer, **(C)** *J-V* characteristics of additive-free OSCs depending on the weight ratio between PTB7-Th and P_Zn_-TNI, and the *J-V* curve of pyridine-treated OSCs at the weight ratio of PTB7-Th:PZn-TNI = 1:1.5 w/w. **(D)** the EQE spectra of PTB7-Th:P_Zn_-TNI devices.

Absorption spectra of P_Zn_-TNI were measured in solution and the film state, as shown in Figure 1. P_Zn_-TNI exhibited clear bimodal absorption composed of the Soret band (300 – 600 nm) and Q-bands (600–800 nm); the maximum absorption peaks of P_Zn_-TNI were 479 and 713 nm in solution, and 478 and 719 nm in the film. The absorption of P_Zn_-TNI was complementary to that of the low-bandgap donor polymer, PTB7-Th, which induced a well-balanced short- and long-wavelength absorption in the entire wavelength of 300–800 nm (Figure 2B). Notably, the film of P_Zn_-TNI showed broadened and red-shifted Q-band absorption spectra compared to that in solution, indicating the enhanced intermolecular π-π stacking in the film state. Since the planar NIs and P_Zn_ are connected by an sp-hybridized ethyne π-bridge, P_Zn_-TNI possesses a highly planar conjugated backbone for efficient intermolecular stacking. The optical bandgap ($E_{\text{g}}^{{}^{\circ}\text{pt}}$) of P_Zn_-TNI was 1.63 eV, which was calculated from the absorption onset wavelength of 761 nm in the film. We previously reported the pyridine additive effect on the molecular ordering of porphyrin derivatives; pyridine enhances the intermolecular ordering of porphyrin derivatives via coordination to the zinc (Hadmojo et al., 2017). As shown in Figure 1B, the absorption of P_Zn_-TNI was significantly broadened and red-shifted compared to that without pyridine treatment, resulting in the bathochromic shift of 42 nm in the Soret band and 15 nm in the Q-band. This supports our previous hypothesis for the pyridine effect on the molecular ordering and confirms that pyridine enhances the intermolecular ordering of P_Zn_-TNI in the film states.

To evaluate the energy levels of P_Zn_-TNI, the highest occupied molecular orbital (HOMO) energy levels and the lowest unoccupied molecular orbital (LUMO) energy levels were measured using cyclic voltammetry. The oxidation and reduction onset potentials of P_Zn_-TNI were 1.07 and −0.80 V, respectively, which corresponds to HOMO levels (*E*_HOMO,CV_) and LUMO levels (*E*_LUMO,CV_) of −5.50 and −3.62 eV, respectively. The optical LUMO energy level (*E*_LUMO,UV_) was calculated to be −3.87 eV from *E*_HOMO,CV_ and $E_{\text{g}}^{{}^{\circ}\text{pt}}$ of P_Zn_-TNI. The energy diagram of the PTB7-Th and P_Zn_-TNI was shown in Figure 1D, and the *E*_HOMO,CV_, *E*_LUMO,CV_, and *E*_LUMO,UV_ of polymer donor (PTB7-Th) were taken from our previous measurement (Zhang et al., 2015; Hadmojo et al., 2016). The LUMO energy level of P_Zn_-TNI is suitable for electron transport from PTB7-Th to P_Zn_-TNI, while the HOMO of P_Zn_-TNI is appropriate for hole transport from P_Zn_-TNI to PTB7-Th (Marcus, 1963; Clarke and Durrant, 2010). The optical and electrochemical properties of P_Zn_-TNI are summarized in Table 1.

**Table 1:** Optical and electrochemical properties of P_Zn_-TNI.

	**UV-Vis absorption**				**Cyclic voltammetry**				
	***^a^*Solution**	*****^b^*******Film**			
	**λ_max_ (nm)**	**λ_max_ (nm)**	**λ_onset_ (nm)**	***^c^*$E_{\text{g}}^{\text{opt}}$ (eV)**	***E*_ox_ (V)**	***E*_red_ (V)**	***^d^E*_HOMO,CV_ (eV)**	***E*_LUMO,CV_ (eV)**	***^e^E*_LUMO,UV_ (eV)**
P_Zn_-TNI	479, 713	478, 719	761	1.63	1.07	−0.80	−5.50	−3.62	−3.87

a Chloroform solution.

b Film on a quartz plate.

c Bandgap calculated from the film-state absorption onset wavelength (λ_onset_).

d HOMO levels determined from E_ox_ of the first oxidation potential of P_Zn_-TNI.

e *LUMO levels calculated from E_HOMO_, _CV_ and ${\text{E}}_{\text{g}}^{{}^{\circ}pt}$*.

Porphyrin acceptor-based fullerene-free OSCs were fabricated by blending PTB7-Th and P_Zn_-TNI (Figure 2A). The current density-voltage (*J-V*) characteristic of PTB7-Th:P_Zn_-TNI devices was investigated via changing the weight ratio between PTB7-Th and P_Zn_-TNI, and the photovoltaic performance was optimized at the weight ratio of 1:1.5 w/w. The photovoltaic properties are summarized in Figure 2C, Figure S6, and Table 2. The best PCE of 5.07% was achieved with a *V*_OC_ of 0.72 V, a *J*_SC_ of 13.84 mA cm^−2^, and FF of 0.51 (Figure 2C). As shown in Figure 2D, the external quantum efficiency (EQE) spectra of PTB7-Th:PZn-TNI devices cover the entire visible area of 300–800 nm and showed the panchromatic photon-to-current conversion due to the complementary solar flux absorption between PTB7-Th and PZn-TNI. Notably, the additive-free film-formation process provided superior photovoltaic performance compared to the pyridine-assisted one as shown in Figure 2C. As shown in Figure 2D, the EQE was increased in the entire wavelength, which indicates that hole/electron transport properties of both PTB7-Th and P_Zn_-TNI are improved in the additive-free devices.

**Table 2:** Photovoltaic properties of PTB7-Th:P_Zn_-TNI devices.

**PTB7-Th:P**_**Zn**_**-TNI**		**μ_h_ (cm^2^ V^−1^·s^−1^)**	**μ_e_ (cm^2^ V^−1^·s^−1^)**	***V*_OC_ (V)**	***J*_SC_ (mA cm^−2^)**	**FF**	**PCE (PCE_ave_)^a^(%)**
No additives	1:1	–	–	0.73	11.25	0.52	4.29 (3.97)
	1:1.5	2.9 × 10^−4^	2.5 × 10^−6^	0.72	13.84	0.51	5.07 (4.85)
	1:2	–	–	0.71	13.81	0.49	4.82 (4.59)
1% (v/v) pyridine	1:1.5	2.4 × 10^−4^	1.3 × 10^−6^	0.78	6.76	0.34	1.82 (1.61)

a *Average PCEs more than 10 devices*.

**Figure 3:**
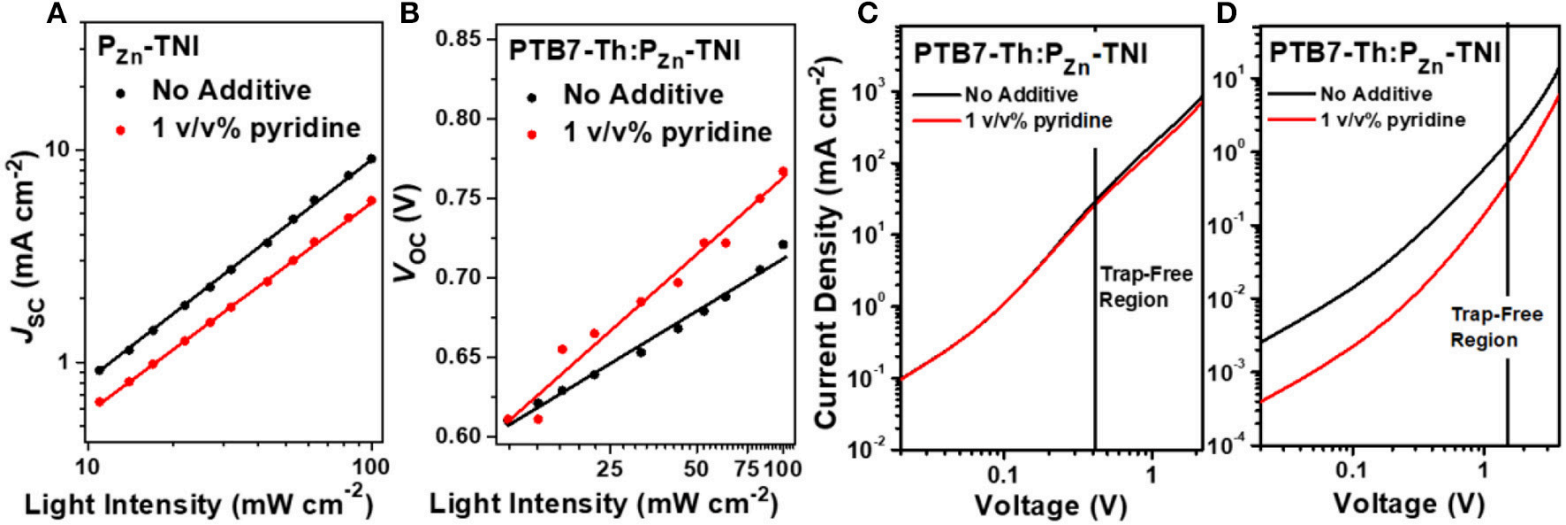
**(A)** *J*_SC_ and **(B)** *V*_OC_ as a function of the illumination intensity. SCLC mobility of **(C)** hole-only and **(D)** electron-only devices.

To understand the charge recombination mechanisms of PTB7-Th:P_Zn_-TNI devices in the presence and absence of pyridine additive, the *J-V* characteristics were investigated as a function of the illumination intensity. The power law dependence of *J*_SC_ on the illumination intensity is generally expressed as *J*_SC_ ∞ *I*^α^, where *I* is the light intensity and α is an exponential factor (Figure 3A) (Blom et al., 2007; Azmi et al., 2016). The α value of the PTB7-Th:P_Zn_-TNI devices was close to unity regardless of additive treatment, indicating the negligible bimolecular recombination in PTB7-Th:P_Zn_-TNI devices. However, the *V*_OC_ vs. illumination intensity was highly affected by the pyridine treatment. Assuming there is no trap-assisted recombination under an open-circuit condition, the slope of *V*_OC_ vs. the illumination intensity produces 1.00 *k*T/*q* (Mihailetchi et al., 2006). The higher value of *k*T/*q* indicates the more probability of trap-assisted recombination under an open-circuit condition (Mandoc et al., 2007; Azmi et al., 2016). As shown in Figure 3B, PTB7-Th:P_Zn_-TNI devices with and without additives show the slope of 2.63 and 1.93 *k*T/*q*, respectively. This implies that PTB7-Th:P_Zn_-TNI devices in the absence of additives have the lowest trap-assisted recombination in anopen-circuit condition. The hole and electron transport properties of PTB7-Th:P_Zn_-TNI devices were measured by a space-charge-limited-current (SCLC) analysis (Figures 3C,D) (Mihailetchi et al., 2005). The electron- and hole-only devices were fabricated with a structure of ITO/ZnO/PTB7-Th:P_Zn_-TNI/ZnO/Al and ITO/PEDOT:PSS/PTB7-Th:P_Zn_-TNI/MoO_x_/Ag, respectively. In the presence of pyridine additive, the hole and electron mobilities of PTB7-Th:P_Zn_-TNI were 2.4 × 10^−4^ and 1.3 × 10^−6^ cm^2^ V^−1^·s^−1^, respectively, whereas, in the absence of additives, the hole and electron mobilities were increased to 2.9 × 10^−4^ and 2.5 × 10^−6^ cm^2^ V^−1^·s^−1^, respectively. Thus, it is expected that the pyridine additive worsens the nanomorphology of PTB7-Th:P_Zn_-TNI devices via excessive intermolecular aggregation.

**Figure 4:**
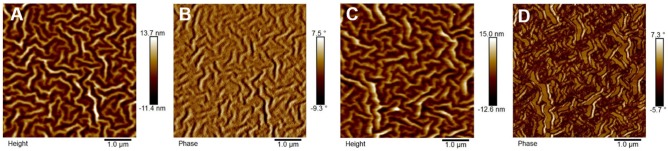
AFM **(A,C)** height and **(B,D)** phase image of PTB7-Th:PZn-TNI; **(A,B)** in the absence of pyridine and **(C,D)** in the presence of pyridine.

**Figure 5:**
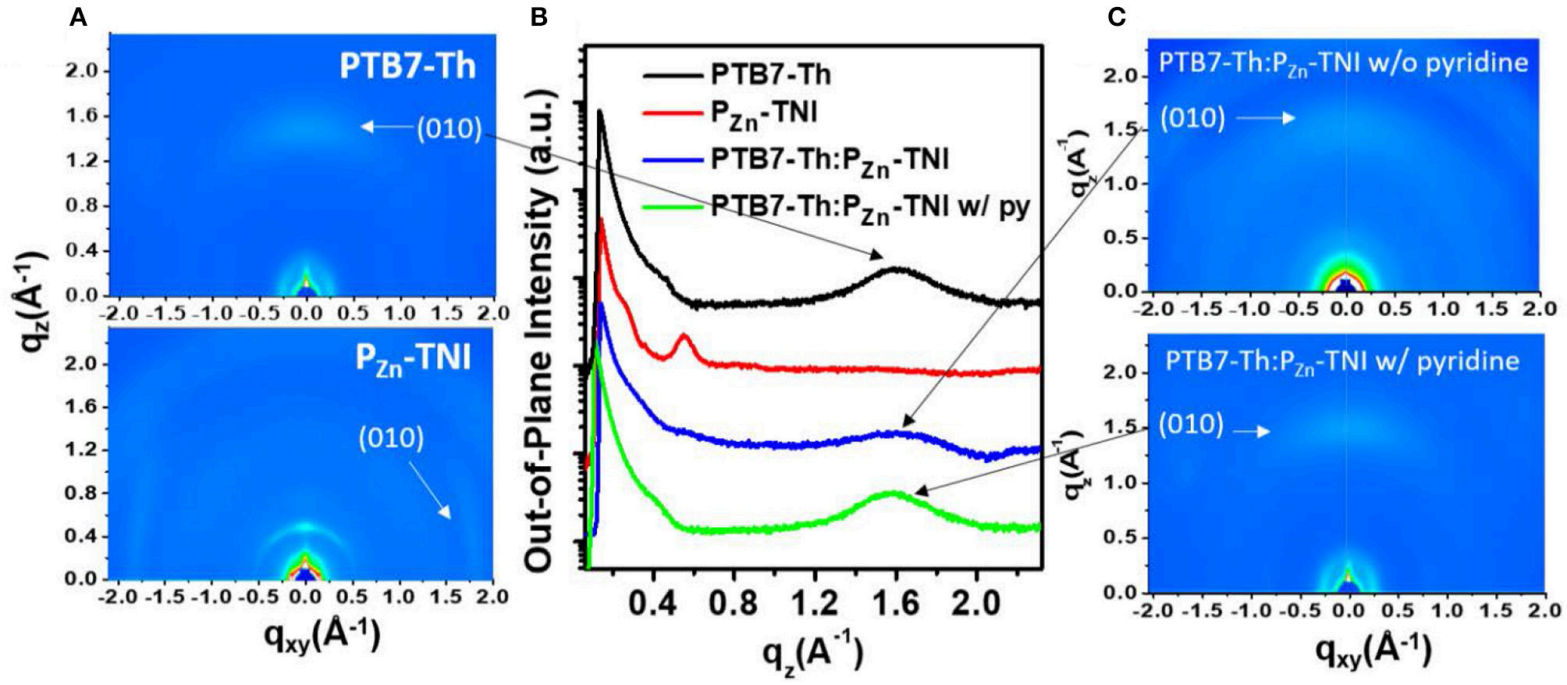
**(A)** 2D-GIXD images of **(A)** pristine film of PTB7-Th and P_Zn_-TNI, and **(C)** additive-free PTB7-Th:P_Zn_-TNI blend film and pyridine-treated one. **(B)** Out-of-plane line-cut of PTB7-Th, P_Zn_-TNI, and PTB7-Th:P_Zn_-TNI film.

The morphology of the PTB7-Th:P_Zn_-TNI active layer was investigated by atomic force microscopy (AFM) (Figure 4) and two-dimensional grazing incidence X-ray diffraction (2D-GIXD) analyses (Figure 5). In AFM images, PTB7-Th:P_Zn_-TNI blended film possesses bicontinuous crystalline domains in the absence of additives (Figures 4A,B), whereas the addition of pyridine additive intensifies the intermolecular ordering of P_Zn_-TNI domains, leading to severe phase segregation between PTB7-Th and P_Zn_-TNI (Figures 4C,D). The 2D-GIXD results also support the AFM analysis. Additive-free PTB7-Th:P_Zn_-TNI film showed clear π-π stacking orientation (010) peak at ~1.6 Å^−1^ along the q_z_ axis, which indicates the face-on orientation with a d-spacing of ~3.9 Å (Figures 5B,C). However, the pyridine-treated PTB7-Th:P_Zn_-TNI film showed the increased π-π stacking interaction and induced the phase aggregation (Figure 5C). The (010) peak in the blend film is assigned to the orientation of PTB7-Th domains (Figure 5A), which implies that the decrease in the photovoltaic performances in presence of pyridine is probably due to the aggregation of the PTB7-Th domains in the PTB7-Th:P_Zn_-TNI blend film. As a result, the P_Zn_-TNI having highly planar molecular structure possesses the sizable π-π intermolecular stacking and crystalline nanomorphology in the additive-free solvent system, which means that no more post-treatment is required in PTB7-Th:PZn-TNI blend system. In addition, the additive-free system can prevent undesirable morphological change and photo-oxidation degradation by additives in the active layer (Li et al., 2017).

## Conclusions

We have synthesized a novel porphyrin acceptor, P_Zn_-TNI, by incorporating four naphthalene imide (NI) units at the meso position of the P_Zn_ core. P_Zn_-TNI showed unique bimodal absorption with a strong Soret band and a weak Q-band. The insufficient long-wavelength absorption of P_Zn_-TNI was covered by a low-bandgap donor, PTB7-Th. As a result, bulk heterojunction fullerene-free OSCs composed of P_Zn_-TNI and PTB7-Th showed panchromatic photon-to-current conversion covering entire area of 300–800 nm. The PTB7-Th:P_Zn_-TNI devices exhibited a promising PCE of 5.07%, which is the highest and the first promising PCE in the porphyrin-based acceptors except for those utilizing the PDI units. Notably, the additive-free solution process provided the best photovoltaic performance, whereas the pyridine additive had a negative effect on the nanomorphology by the excessive molecular aggregation of the PTB7-Th:P_Zn_-TNI film. The planar backbone structure of P_Zn_-TNI assists the sizable molecular ordering in the PTB7-Th:P_Zn_-TNI film without additive treatment, which is favorable for practical applications.

## Author contributions

IJ and SY conceived the ideas and designed the PZn-TNI. U-HL synthesized all the materials and JK assisted the characterization of all the materials. S-YJ supervised all the device fabrication and optimization. WH fabricated all the OSC devices and SE assisted the device characterization.

### Conflict of interest statement

The authors declare that the research was conducted in the absence of any commercial or financial relationships that could be construed as a potential conflict of interest.
