# Supplementary material for: Development of n-Type Porphyrin Acceptors for Panchromatic Light-Harvesting Fullerene-Free Organic Solar Cells
Source: Front Chem. 2018 Oct 9;6:473. doi: 10.3389/fchem.2018.00473 (PMC6189314; doi:10.3389/fchem.2018.00473)
Supplement: Supplementary file 1 [file Data_Sheet_1.pdf]

frontiers

Supplementary Material

Development of n-Type Porphyrin Acceptors for Panchromatic Light-Harvesting Fullerene-Free Organic Solar Cells

Un-Hak Lee[1], Wisnu Tantyo Hadmojo[2], Junho Kim[2], Seung Hun Eom[1], Sung Cheol Yoon[1*], Sung-Yeon Jang[2*], In Hwan Jung[2*]

[1]Division of Advanced Materials, Korea Research Institute of Chemical Technology (KRICT), 141 Gajeong-ro, Yuseong-gu, Daejeon 34114, Republic of Korea.

[2]Department of Chemistry, Kookmin University, 77 Jeongneung-ro, Seongbuk-gu, Seoul 02707, Republic of Korea.

*** Correspondence:**
Sung Cheol Yoon: yoonsch@krict.re.kr
Sung-Yeon Jang: syjang@kookmin.ac.kr
In Hwan Jung: ihjung@kookmin.ac.kr

1 Experimental

1.1 Device fabrication

ITO substrates were pre-cleaned by ultrasonication in acetone and isopropyl alcohol (IPA), each for 20 minutes. Bulk heterojunction devices were fabricated with a structure configuration of ITO/ZnO/PTB7-Th:P_{Zn}-TNI/MoOx/Ag. Electron transporting layers of ZnO (~30 nm) were prepared by in-situ conversion. Zinc acetate dehydrate ([$Zn(CH_3COO)_2 \cdot 2H_2O$], Sigma Aldrich, 99.99 %, 5 g) was used as precursor, and ethanolamine ($NH_2CH_2CH_2OH$, Sigma Aldrich, 99.5 %, 1.35 mL) in 2-methoxyethanol ($CH_3OCH_2CH_2OH$, Sigma Aldrich, 99.8 %, 50 mL) was spin-coated at 4000 rpm for 15 s, followed by thermal annealing gradually from room temperature to 200 °C for 10 minutes. The active layer of PTB7-Th:P_{Zn}-TNI was prepared in a glove box by spin coating (~100 nm). The weight ratio of donor:acceptor was 1:1.5 w/w. Chloroform was used as solvent, and the active layer materials were stirred for 2 h. The total concentration was 15 mg/mL. MoOx (8 nm) as electron blocking layer and Ag (150 nm) as metal electrode, were deposited by thermal evaporation under reduced pressure (10^{-6} bar).

1.2 Device analysis

The *J-V* characteristics of the BHJ devices were measured using a Keithley 2401 instrument and a solar simulator with a 150 W Xenon lamp (Newport) as light source. The light source was calibrated to AM 1.5 G using a monosilicon standard from the National Renewable Energy Laboratory (NREL). The EQE was measured by passing the 400 W Xenon lamp light source to a monochromator using an appropriate wavelength filter (Mc Science, K3100 IQX). The chopping frequency was 5 Hz. Space charge limited current (SCLC) measurements was done in the dark. A hole-only device (ITO/PEDOT:PSS/PTB7-Th:P_{Zn}-TNI/MoOx/Ag) and electron-only device (ITO/ZnO/PTB7-Th:P_{Zn}-TNI/ZnO/Al) were used to measure the hole and electron mobility of the devices. The mobility was calculated using the Mott-Gurney law in the SCLC trap-free regime.

1.3 Sample characterization

All commercially available reagents were reagent grade and used without further purification. Dichloromethane (CH_2Cl_2), tetrahydrofuran (THF), and *N*,*N*-Dimethylformamide (DMF) were freshly distilled before each use. The ^1H NMR spectra were recorded on a Bruker (Bruker Avance III HD 400 or Avance NEO) spectrometer in $CDCl_3$ (25 °C) and THF-d_8 (25 °C). MALDI-TOF-MS was performed on a Bruker Daltonics LRF20 with dithranol (1,8,9-trihydroxyanthracene) as the matrix. Recycling SEC was performed on a JAI model LC9021 equipped with JAIGEL-1H, JAIGEL-2H, and JAIGEL-3H columns using THF (DUKSAN) as the eluent. Cyclic voltammetry was performed on a BAS 100B/W electrochemical analyzer with a three-electrode cell in a 0.1N Bu_4NBF_4 solution in acetonitrile at a scan rate of 50 mV s^{-1}. The P_{Zn}-TNI film was coated onto a Pt wire electrode by dipping the electrode into P_{Zn}-TNI solution in chloroform. All measurements were calibrated against an internal standard of ferrocene (Fc), the ionization potential (IP) value (which is −4.8 eV for the Fc/Fc^+ redox system). The absorption spectra were measured on a SHIMADZU/UV-2550 model UV-visible spectrophotometer. The morphology of the blend films was measured using atomic force microscopy (AFM) with tapping mode on a Nanoscope instrument (Bruker).

1.4 Two-Dimensional Grazing-Incidence X-ray Diffraction (2D-GIXRD)

2D-GIXRD measurements were performed at the 9A beamline in the Pohang Accelerator Laboratory (PAL), Korea. The samples were prepared on top of Si substrates. The beam energy was 11.025 keV, and the distance from the sample to the detector was 221 mm. The irradiation time was 5 s and the incident angle was set at 0.12°.

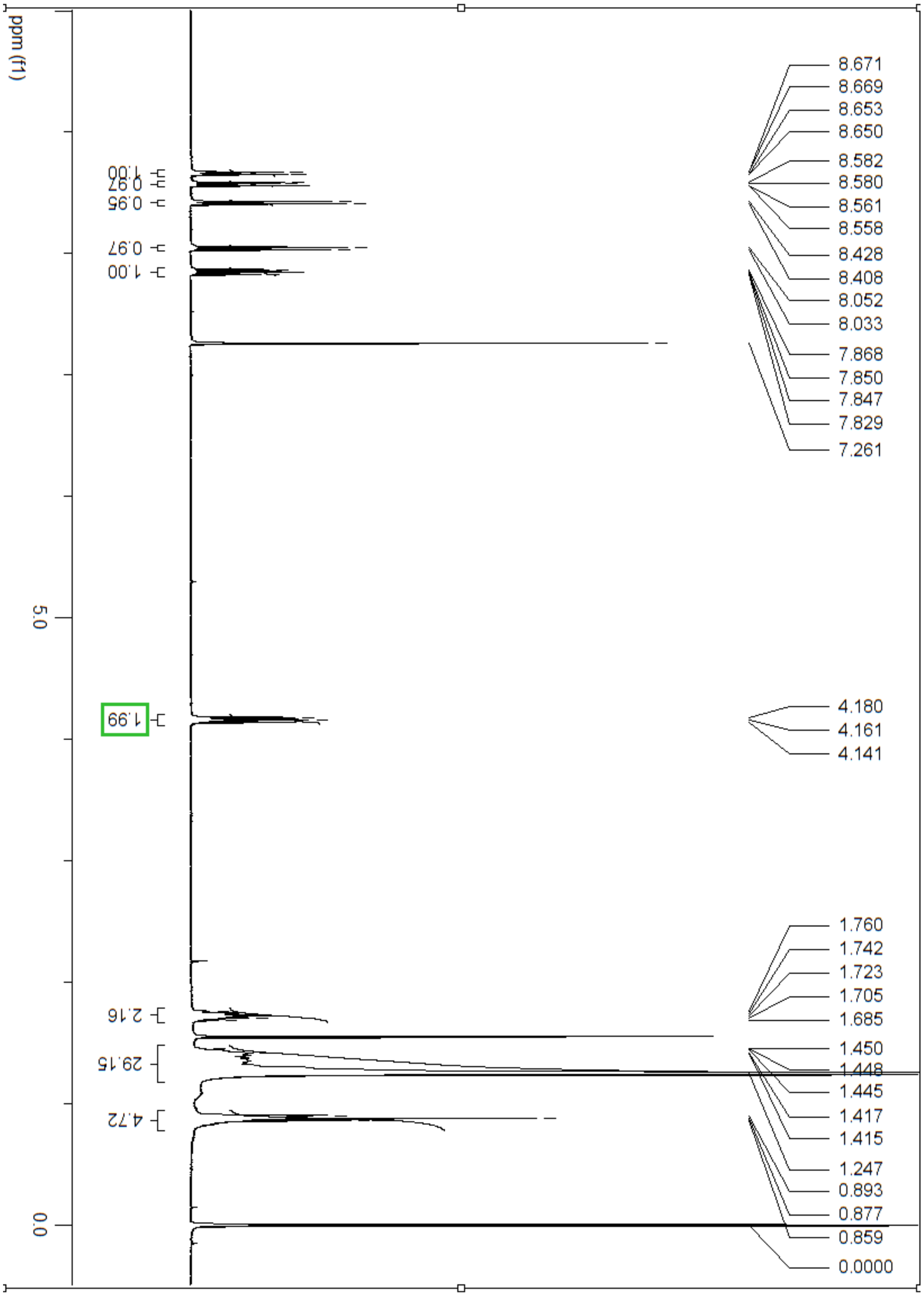

Figure S1. ^{1}H NMR spectrum of compound 4.

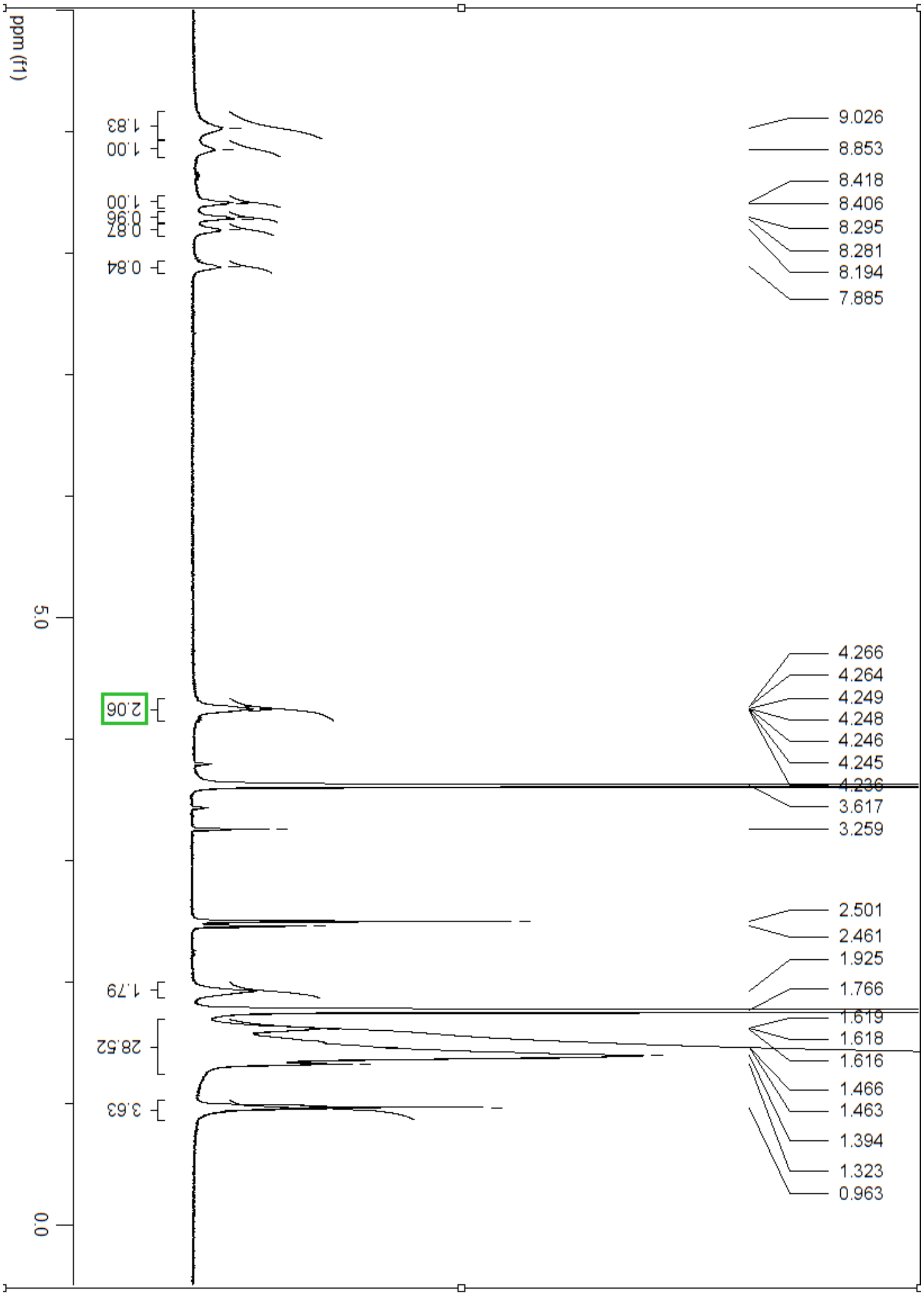

Figure S2. ^{1}H NMR spectrum of P_{Zn}-TNI.

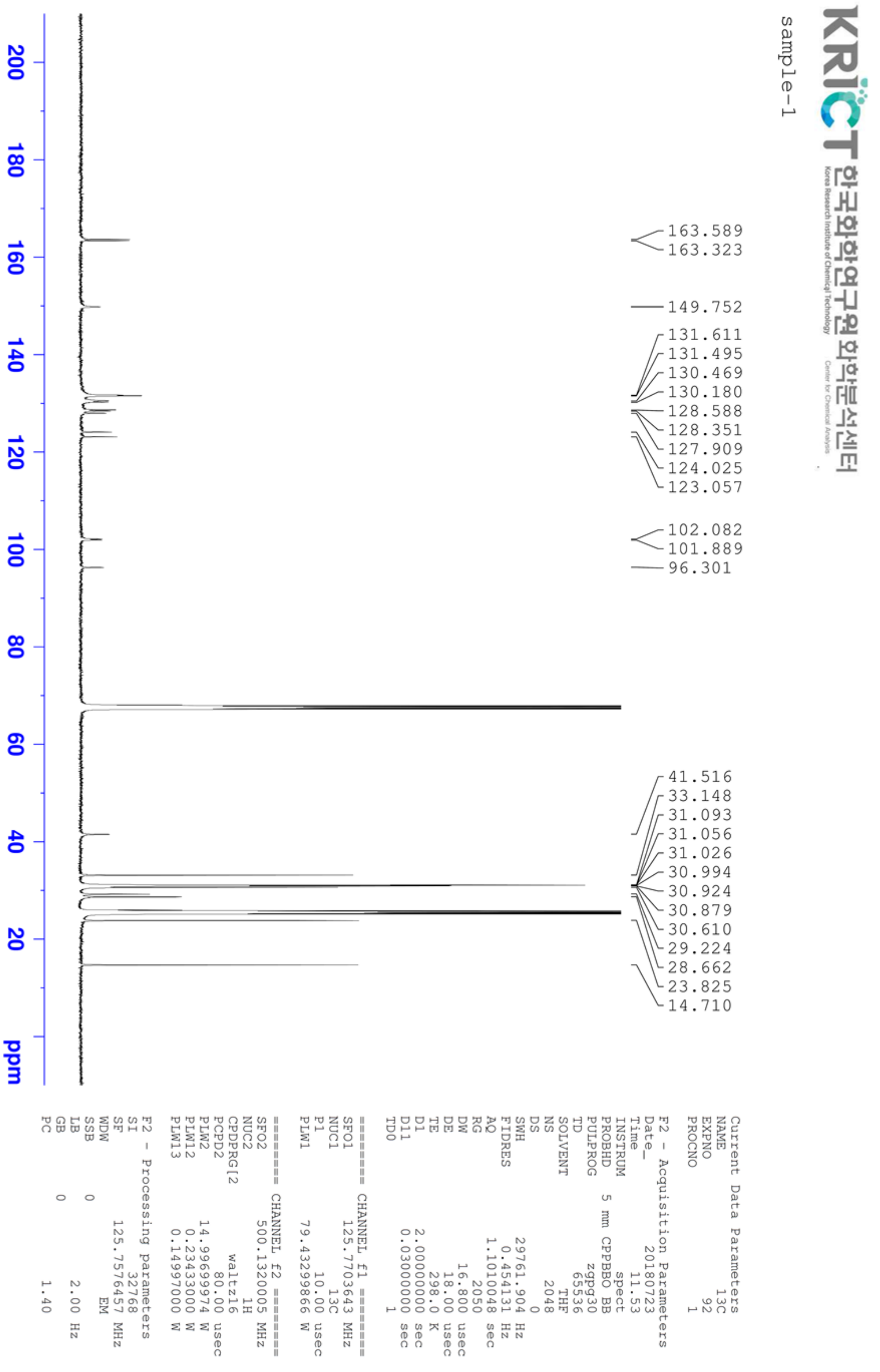

Figure S3. ^{13}C NMR spectrum of P_{Zn}-TNI.

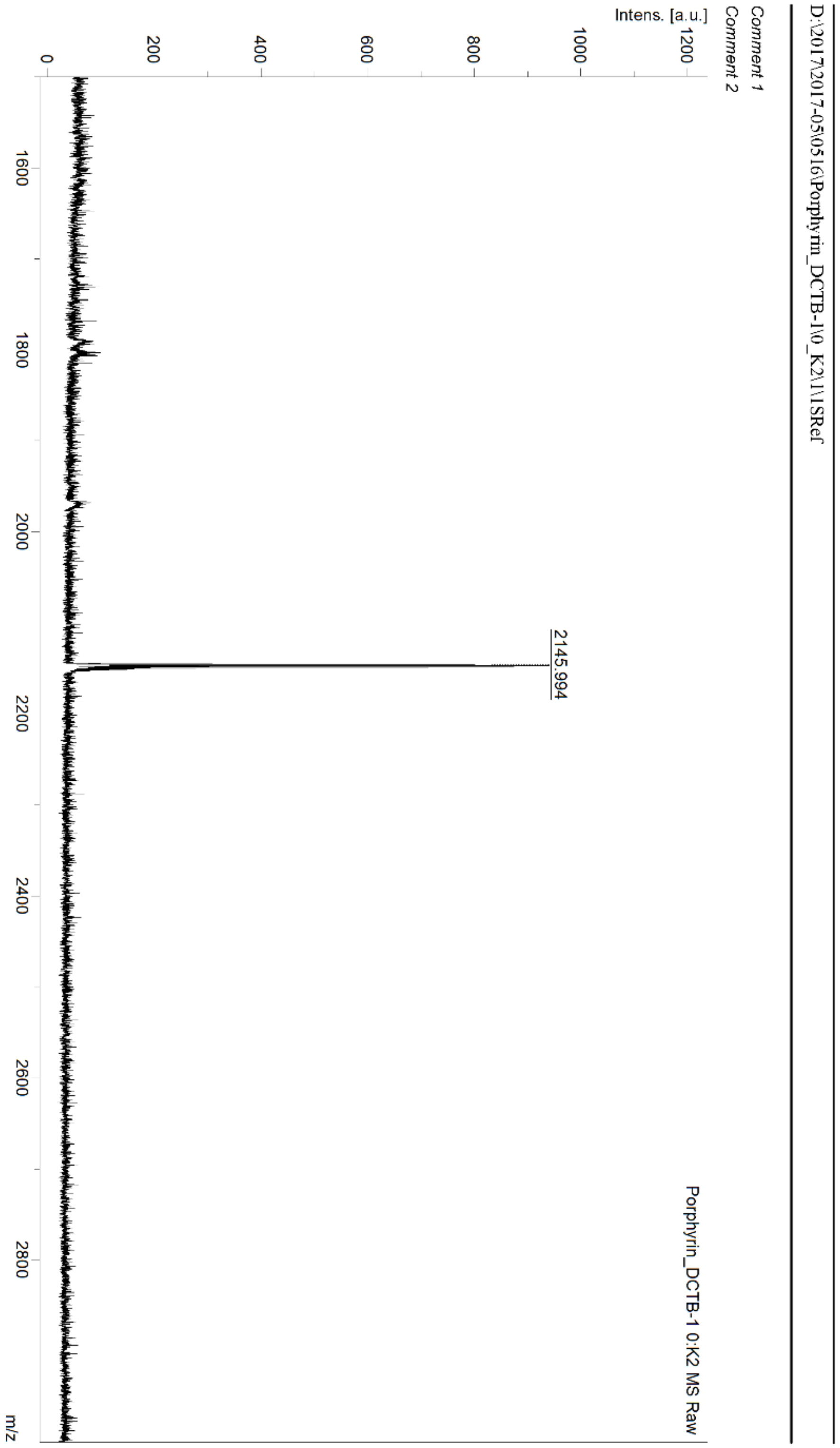

Figure S4. MALDI-TOF-MS spectrum of P_{Zn}-TNI.

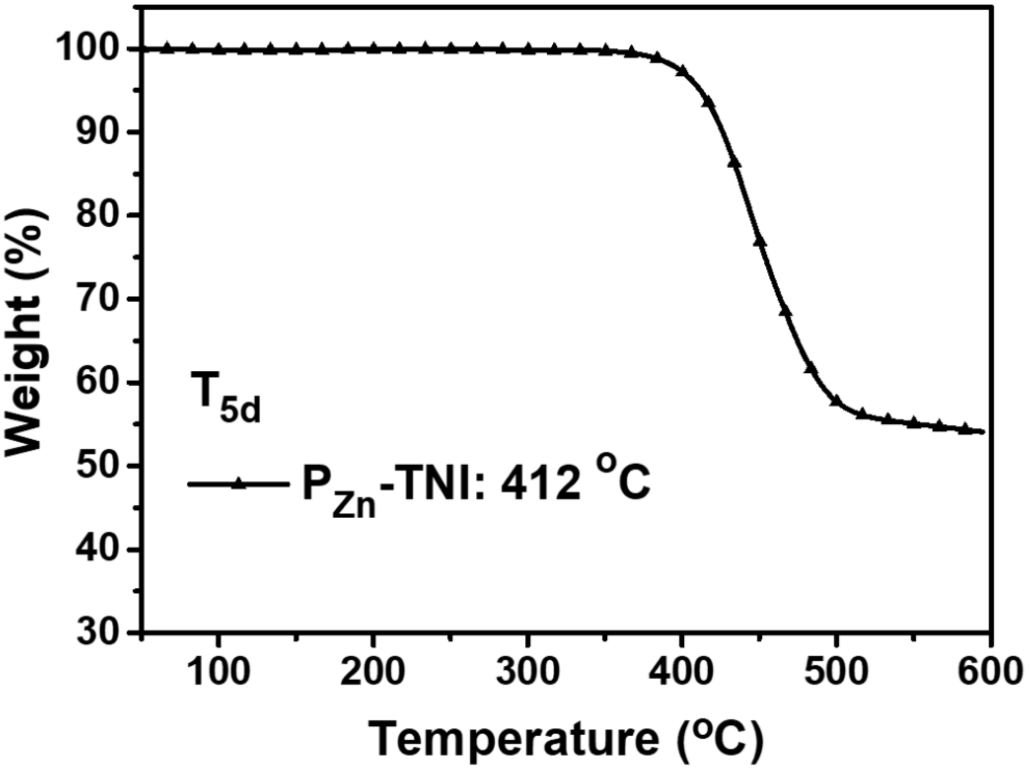

Figure S5. TGA thermogram of P_{Zn}-TNI.

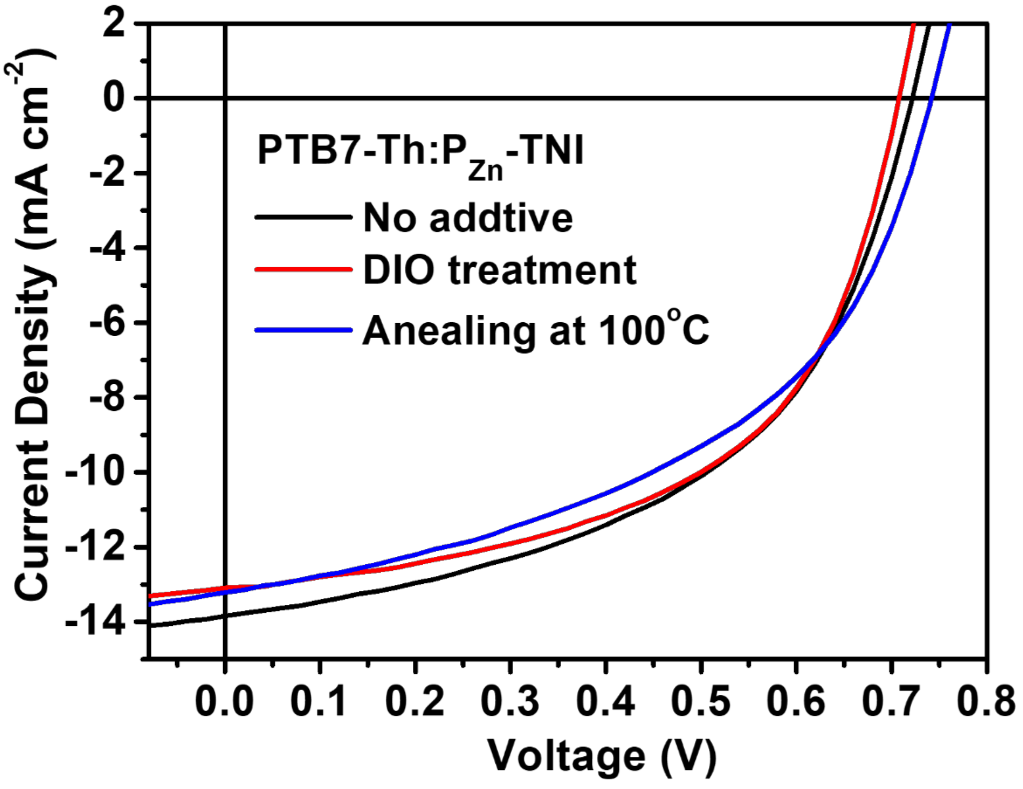

Figure S6. *J*-*V* characteristic of PTB7-Th:P_{Zn}-TNI devices with 1% (v/v) DIO and thermal annealing at 100 °C for 10 min

Table S1. Photovoltaic properties of PTB7-Th:P_{Zn}-TNI devices at the optimum condition

	V_{OC} (V)	J_{SC} (mA cm^{-2})	FF	PCE (%)
PTB7-Th:P_{Zn}-TNI (1:1.5 w/w)	0.71	13.37	0.5	4.75
	0.71	13.66	0.49	4.81
	0.72	13.84	0.51	5.07
	0.71	13.28	0.51	4.83
	0.71	13.5	0.52	4.95
	0.72	13.64	0.51	5.03
	0.71	12.58	0.52	4.71
	0.72	12.71	0.53	4.84
	0.73	12.74	0.53	4.91
	0.71	13.98	0.46	4.6

Table S2. Photovoltaic properties of PTB7-Th:P_{Zn}-TNI devices after post-treatment.

PTB7-Th:P_{Zn}-TNI (1:1.5 w/w)	V_{OC} (V)	J_{SC} (mA cm^{-2})	FF	PCE (%)
N/A	0.72	13.84	0.51	5.07
1 vol% DIO	0.70	13.09	0.54	5.02
Annealing at 100 °C for 10 min	0.74	13.22	0.48	4.70
